# Grain Foods Are Contributors of Nutrient Density for American Adults and Help Close Nutrient Recommendation Gaps: Data from the National Health and Nutrition Examination Survey, 2009–2012

**DOI:** 10.3390/nu9080873

**Published:** 2017-08-14

**Authors:** Yanni Papanikolaou, Victor L. Fulgoni

**Affiliations:** 1 Nutritional Strategies, Inc., 59 Marriott Place, Paris, ON N3L 0A3, Canada; 2 Nutrition Impact, LLC, 9725 D Drive North, Battle Creek, MI 49014, USA; vic3rd@aol.com

**Keywords:** NHANES, energy, Dietary Guidelines, adults, grains, shortfall nutrients

## Abstract

The 2015–2020 Dietary Guidelines for Americans (2015-2020 DGA) maintains recommendations for increased consumption of whole grains while limiting intake of enriched/refined grains. A variety of enriched grains are sources of several shortfall nutrients identified by 2015-2020 DGA, including dietary fiber, folate, iron, and magnesium. The purpose of this study was to determine food sources of energy and nutrients for free-living U.S. adults using data from the National Health and Nutrition Examination Survey, 2009–2012. Analyses of grain food sources were conducted using a single 24-h recall collected in adults ≥19 years of age (*n* = 10,697). Sources of nutrients contained in all grain foods were determined using United States Department of Agriculture nutrient composition databases and the food grouping scheme for grains (excluding mixed dishes). Mean energy and nutrient intakes from the total diet and from various grain food groups were adjusted for the sample design using appropriate weights. All grains provided 285 ± 5 kcal/day or 14 ± 0.2% kcal/day in the total diet in adult ≥19 years of age. In the total daily diet, the grain category provided 7.2 ± 0.2% (4.9 ± 0.1 g/day) total fat, 5.4 ± 0.2% (1.1 ± 0.03 g/day) saturated fat, 14.6 ± 0.3% (486 ± 9 mg/day) sodium, 7.9 ± 0.2% (7.6 ± 0.2 g/day) total sugar, 22.8 ± 0.4% (3.9 ± 0.1 g/day) dietary fiber, 13.2 ± 0.3% (122 ± 3 mg/day) calcium, 33.6 ± 0.5% (219 ± 4 mcg dietary folate equivalents (DFE)/day) folate, 29.7 ± 0.4% (5.3 ± 0.1 mg/day) iron, and 13.9 ± 0.3% (43.7 ± 1.1 mg/day) magnesium. Individual grain category analyses showed that breads, rolls and tortillas and ready-to-eat cereals provided minimal kcal/day in the total diet in men and women ≥19 years of age. Similarly, breads, rolls and tortillas, and ready-to-eat cereals supplied meaningful contributions of shortfall nutrients, including dietary fiber, folate and iron, while concurrently providing minimal amounts of nutrients to limit. Cumulatively, a variety of grain food groups consumed by American adults contribute to nutrient density in the total diet and have the potential to increase consumption of shortfall nutrients as identified by 2015–2020 DGA, particularly dietary fiber, folate, and iron.

## 1. Introduction

Grain foods collectively, whether through enrichment and/or fortification practices, are an integral part of American dietary practices. Grains represent a key component of energy and nutrients in the 2015–2020 Dietary Guidelines for Americans (2015–2020 DGA) dietary patterns, such that at the 2000 calorie level, both the Healthy U.S.- and Healthy Mediterranean-Style patterns recommend six ounce-equivalent (oz. eq.) servings, daily, of grains, with half of those servings being whole grains, while the Healthy Vegetarian eating pattern recommends 6.5 oz. eq. daily servings of grains, with 3.5 oz. eq. stemming from whole grains [1]. The 2015–2020 DGA places emphasis on increasing consumption of whole grains and concurrently limiting consumption of refined and/or enriched grain foods. While certain grains foods may contain higher levels of nutrients to limit, including added sugar, saturated fat, and sodium, many grain foods contribute positive nutrition to the American diet, and are important contributors of the shortfall nutrients. Recently, secondary analyses of the National Health and Nutrition Examination Survey (NHANES) 2005–2010 identified several grain food patterns of consumption in U.S. adults and reported an association between grain food consumption and nutrient intakes, such that several grain food patterns were linked with greater nutrient intakes, including higher intake of shortfall nutrients and nutrients of public health concern as identified by the 2015–2020 DGA [2]. Similarly, certain grain food patterns in children and adolescents, including both whole and refined grains were associated with greater intakes of shortfall nutrients and/or nutrients of concern, including iron, magnesium, vitamin D, dietary fiber, and folate when compared to children and adolescents consuming non-grain dietary patterns [3].

Classifying all grains that are not whole grains into the one category of refined grains may not be a justified representation of the nutrient contribution provided by many nutrient-dense enriched grain foods, including breads, rolls, cooked cereals, and ready-to-eat cereals. Since less than 5% of Americans consume the minimum recommended amount of whole grains, with the average American consuming less than one oz. eq. of whole grains per day [1,4], it can be assumed that the predominant type of grains consumed by Americans, as collected by NHANES, are refined/enriched grains. Recent analyses using NHANES 2009–2012 demonstrated that grain foods are contributors of the 2015–2020 DGA underconsumed nutrients and nutrients of public health concern [5], including dietary fiber, folate, magnesium, calcium, and iron. When considering sub-categories of grain foods, breads, rolls and tortillas, and ready-to-eat cereals were meaningful contributors (i.e., ≥10% in the diet) of dietary fiber, thiamin, folate, iron, zinc and niacin to the American diet of children and adolescents.

The nutritional contribution of specific grain foods in the diet of American adults by gender is limited, thus, the objective of the present analyses was to determine sources of energy and nutrients, with particular focus on shortfall nutrients, from commonly-consumed grain foods in U.S. adults using data from NHANES, 2009–2012.

## 2. Experimental Section

The current analyses were recently completed in children and adolescents [5] and the adult data followed a similar statistical procedure using data from NHANES, a nationally-representative, cross-sectional survey of free-living, non-institutionalized, civilian U.S. residents. NHANES data are collected by the National Center for Health Statistics of the Centers for Disease Control and Prevention. Written informed consent was obtained for all participants or proxies, and the survey protocol was approved by the Research Ethics Review Board at the National Center for Health Statistics. Two NHANES datasets (2009–2010; 2011–2012) were combined for the present analyses [6,7]. Nutrient intake data for NHANES 2009–2012 are from the United States Department of Agriculture (USDA) Food and Nutrient Database for Dietary Studies (FNDDS) 5.0 [8]. FNDDS is a database that provides the nutrient values for foods and beverages reported in What We Eat in America (WWEIA), the dietary intake component of NHANES. The WWEIA Food Categories provide an application to analyze food and beverages as consumed in the American diet. For the current analyses, each of the grain foods reported in WWEIA/NHANES are placed in one of the mutually exclusive food categories by linking each food code contained in FNDDS to one WWEIA category [9]. This food code classification scheme includes approximately 150 unique food categories. Mixed grain dishes were separated from the main grain group and were not included in the current analyses.

In the present analyses, the combined sample included 10,697 male and female adult participants, ≥19 years of age, who had reliable and complete 24-h dietary intake data from WWEIA. Females who reported being pregnant at the time of the NHANES data collection procedure have been excluded from the present analyses.

### Methods and Statistical Analysis

All statistical analyses were performed using SAS software (version 9.2, SAS Institute, Cary, NC, USA). SAS PROC SQL was used to create and prepare data for analyses. SAS PROC SURVEYMEANS was used for all statistical calculations, including means and percentages. Survey weights were used to generate nationally-representative estimates for U.S. adults, which were adjusted for the complex sample design of NHANES. Mean and standard errors of energy, macro-, and micro-nutrients for the daily total diet and from grain food groups were determined. The mean percentage of energy and nutrients contributed from each grain food group were also determined in addition to mean total energy and nutrient intakes. Meaningful sources of energy and nutrients from the overall grain category and the various sub-categories of grain products were defined as an energy or nutrient contribution of ≥10% of the total diet per day. A red solid line was incorporated in all figures to indicate the percentage of energy provided by the grain food category of interest. Those nutrients above the red line show that the grain food category provides a greater percentage in the diet relative to energy, thus indicating nutrient density.

## 3. Results

In addition to an all grain category, six main grain food groups were identified from NHANES and categorized by mean gram weight consumption in the total diet (Table 1).

**Figure 1:**
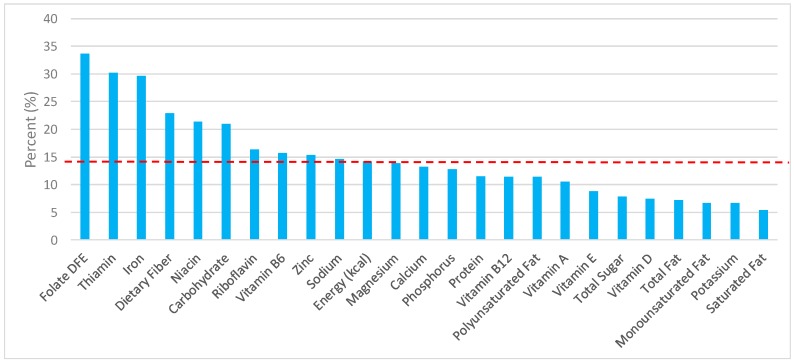
Grain foods sources of energy and nutrients using the National Health and Nutrition Examination Survey (NHANES) 2009–2012, for adults. Data are for all adults ≥19 years of age (*n* = 10,697; males and females), Day 1 intakes; dashed red line represents the percentage of energy provided and nutrients that surpass energy (kcal) contribution. Thus, nutrients above the dashed red line show the food category provides a greater percentage in the diet to showcase meaningful nutrient density in the diet per day. DFE, dietary folate equivalents.

**Figure 2:**
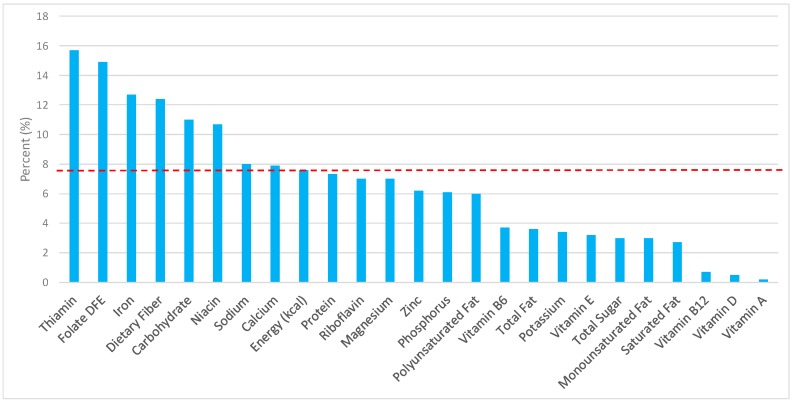
Breads, rolls, and tortillas as sources of energy and nutrients using NHANES 2009–2012, for adult females. Data are for adult females aged ≥19 years of age (*n* = 5349), Day 1 intakes; dashed red line represents the percentage of energy provided and nutrients that surpass energy (kcal) contribution. Thus, nutrients above the dashed red line show the food category provides a greater percentage in the diet to showcase meaningful nutrient density in the diet per day.

**Figure 3:**
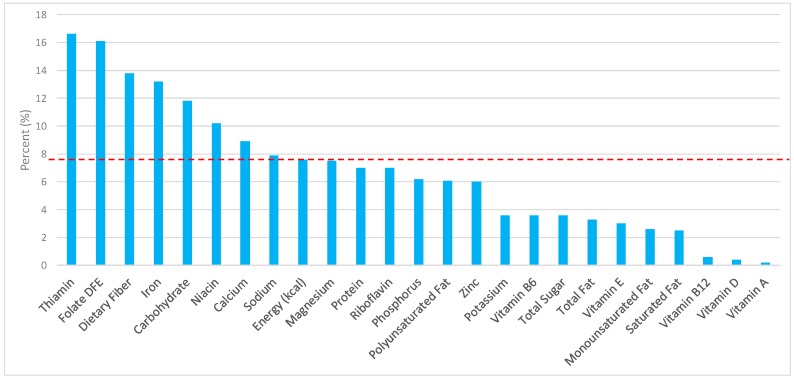
Breads, rolls, and tortillas as sources of energy and nutrients using NHANES 2009–2012, for adult males. Data are for adult males aged ≥19 years of age (*n* = 5348), Day 1 intakes; dashed red line represents the percentage of energy provided and nutrients that surpass energy (kcal) contribution. Thus, nutrients above the dashed red line show the food category provides a greater percentage in the diet to showcase meaningful nutrient density in the diet per day.

**Figure 4:**
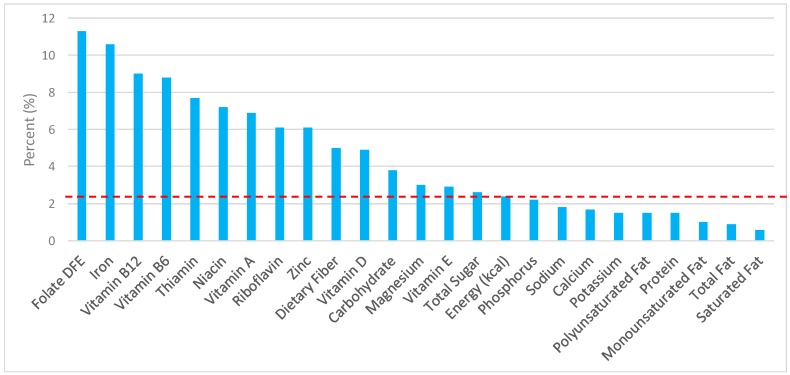
Ready-to-eat cereals as sources of energy and nutrients using NHANES 2009–2012, for adult females. Data are for adult females aged ≥19 years of age (*n* = 5349), Day 1 intakes; dashed red line represents the percentage of energy provided and nutrients that surpass energy (kcal) contribution. Thus, nutrients above the dashed red line show the food category provides a greater percentage in the diet to showcase meaningful nutrient density in the diet per day.

**Figure 5:**
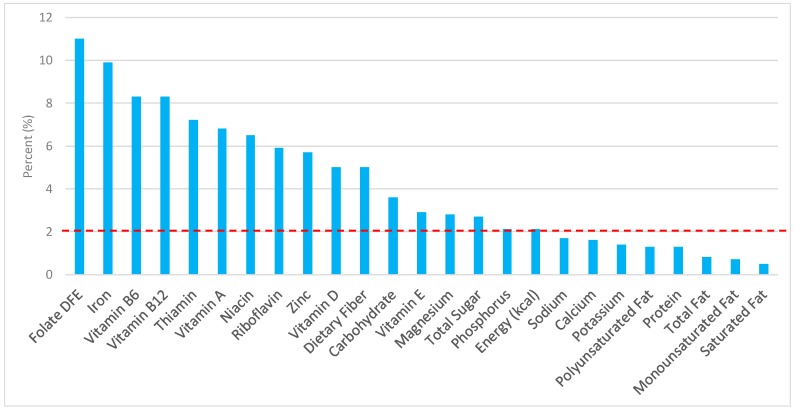
Ready-to-eat cereals as sources of energy and nutrients using NHANES 2009–2012, for adult males. Data are for adult males aged ≥19 years of age (*n* = 5348), Day 1 intakes; dashed red line represents the percentage of energy provided and nutrients that surpass energy (kcal) contribution. Thus, nutrients above the dashed red line show the food category provides a greater percentage in the diet to showcase meaningful nutrient density in the diet per day.

**Figure 6:**
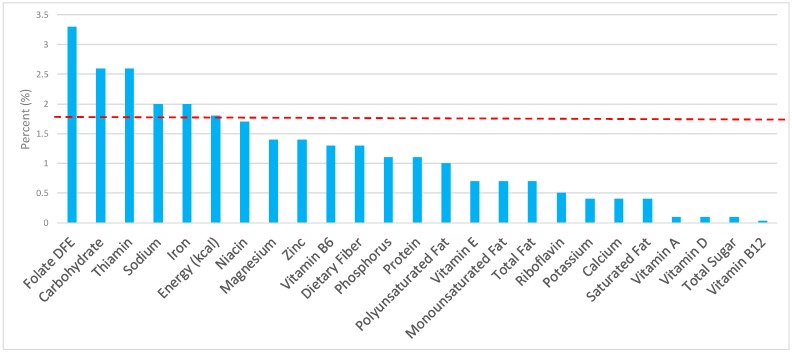
Cooked grains as sources of energy and nutrients using NHANES 2009–2012, for adult females. Data are for adult females aged ≥19 years of age (*n* = 5349), Day 1 intakes; dashed red line represents the percentage of energy provided and nutrients that surpass energy (kcal) contribution. Thus, nutrients above the dashed red line show the food category provides a greater percentage in the diet to showcase meaningful nutrient density in the diet per day.

**Figure 7:**
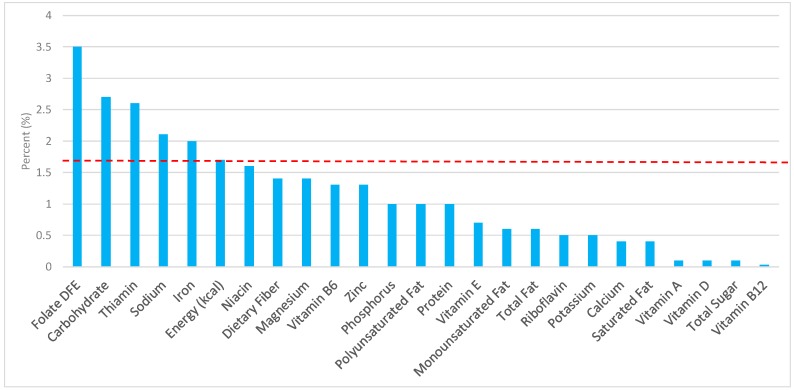
Cooked cereals as sources of energy and nutrients using NHANES 2009–2012, for adult males. Data are for adult males aged ≥19 years of age (*n* = 5348), Day 1 intakes; dashed red line represents the percentage of energy provided and nutrients that surpass energy (kcal) contribution. Thus, nutrients above the dashed red line show the food category provides a greater percentage in the diet to showcase meaningful nutrient density in the diet per day.

**Figure 8:**
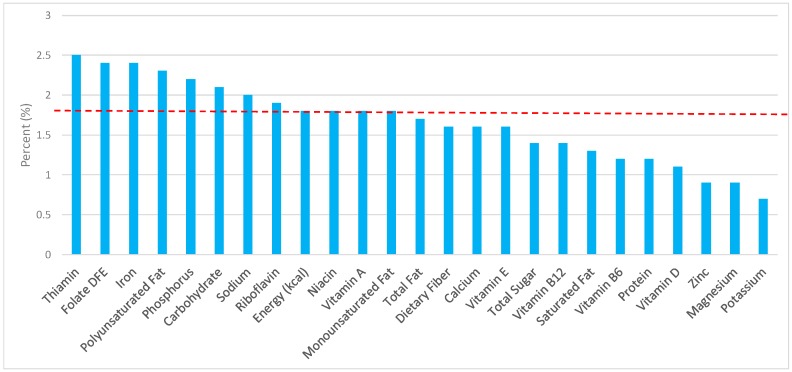
Quick breads and bread products as sources of energy and nutrients using NHANES 2009–2012, for adult females. Data are for adult females aged ≥19 years of age (*n* = 5349), Day 1 intakes; dashed red line represents the percentage of energy provided and nutrients that surpass energy (kcal) contribution. Thus, nutrients above the dashed red line show the food category provides a greater percentage in the diet to showcase meaningful nutrient density in the diet per day.

**Figure 9:**
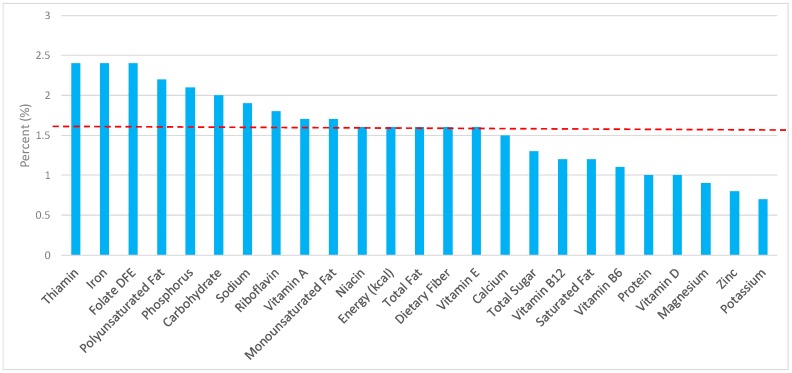
Quick breads and bread products as sources of energy and nutrients using NHANES 2009–2012, for adult males. Data are for adult males aged ≥19 years of age (*n* = 5348), Day 1 intakes; dashed red line represents the percentage of energy provided and nutrients that surpass energy (kcal) contribution. Thus, nutrients above the dashed red line show the food category provides a greater percentage in the diet to showcase meaningful nutrient density in the diet per day.

**Figure 10:**
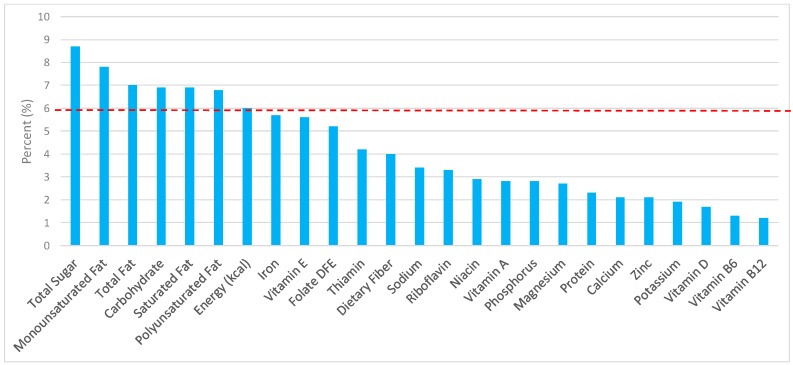
Sweet bakery products as sources of energy and nutrients using NHANES 2009–2012, for females. Data are for adult females aged ≥19 years of age (*n* = 5349), Day 1 intakes; dashed red line represents the percentage of energy provided and nutrients that surpass energy (kcal) contribution. Thus, nutrients above the dashed red line show the food category provides a greater percentage in the diet to showcase meaningful nutrient density in the diet per day.

**Figure 11:**
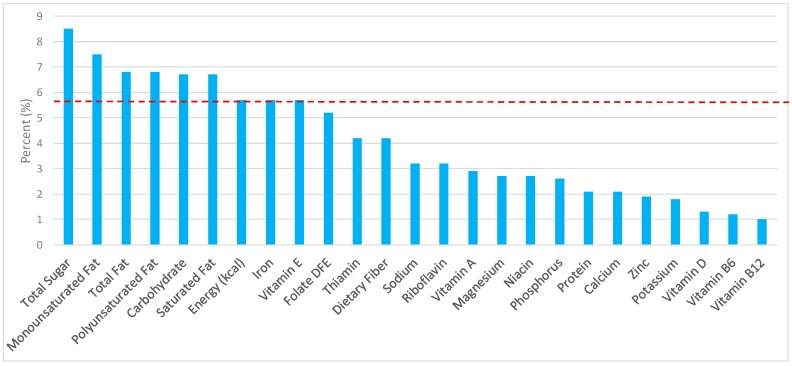
Quick breads and bread products as sources of energy and nutrients using NHANES 2009–2012, for adult males. Data are for adult males aged ≥19 years of age (*n* = 5348), Day 1 intakes; dashed red line represents the percentage of energy provided and nutrients that surpass energy (kcal) contribution. Thus, nutrients above the dashed red line show the food category provides a greater percentage in the diet to showcase meaningful nutrient density in the diet per day.

The various grain food sources of energy and nutrients are shown in Figure 1, Figure 2, Figure 3, Figure 4, Figure 5, Figure 6, Figure 7, Figure 8, Figure 9, Figure 10 and Figure 11. Energy and 24 nutrients are listed from all grain foods and various subsets within commonly consumed grain foods (i.e., breads, rolls, tortillas, ready-to-eat cereals, cooked grains, etc.), of which include nutrients to limit and encourage in the daily diet as outlined by dietary guidance [1].

### 3.1. All Grain Foods: Sources of Energy and Nutrients

Percentage of total intake of energy and nutrients contributed from the total grain food category in both genders, excluding mixed grain dishes, can be seen in Figure 1. All grain foods contributed 14.6% of sodium, 14.2% of energy, 7.9% of total sugar, 7.2% of total fat, 5.4% of saturated fat for nearly a quarter of total dietary fiber (22.9%), 33.6% of folate, 29.7% of iron, 10.6% of vitamin A, 13.9% of magnesium, and 13.2% of calcium per day. Dietary fiber, folate, iron, vitamin A, magnesium and calcium have been identified as shortfall nutrients by the 2015–2020 DGA [1]. Grains also provided 30.2% of thiamin, 21.4% of niacin, 16.4% of riboflavin, 15.7% of vitamin B6, 15.4% of zinc, 11.4% of vitamin B_12_, and 12.8% of phosphorus in the adult diet. Thus, all grain foods contributed less than 15% of all calories in the total diet, while delivering greater than 20% of intake for three shortfall nutrients (dietary fiber, folate, iron), and greater than 10% of intake for three additional shortfall nutrients (calcium, magnesium, vitamin A).

### 3.2. Breads, Rolls, and Tortillas: Sources of Energy and Nutrients by Gender

When examining the adult female population, the percentage of total intake of energy and nutrients contributed from the bread, rolls, and tortilla category, excluding mixed grain dishes can be seen in Figure 2. When considering nutrients to limit, breads, rolls, and tortillas contributed 8.0% of sodium, 3.0% of total sugar, 3.6% of total fat, and 2.7% of saturated fat, while providing 7.6% of total energy in the daily diet of adult females. Breads, rolls, and tortillas were responsible for approximately 12.4% of total dietary fiber, 14.9% of folate, 12.7% of iron, 7.9% of calcium, and 6.8% of magnesium per day, all nutrients identified as shortfall nutrients by the 2015–2020 DGA [1]. Additionally, breads, rolls, and tortillas contributed 15.7% of thiamin, 10.7% niacin, 7% riboflavin, and 6.2% of zinc daily.

Similar findings were seen in adult males (Figure 3)—7.9% of sodium, 3.6% of total sugar, 3.3% of total fat, 2.5% of saturated fat, and 7.6% of total energy in the daily diet stemmed from breads, rolls, and tortillas. For shortfall nutrients identified by the 2015–2020 DGA [1], breads, rolls and tortillas contributed approximately 13.8% of total dietary fiber, 16.1% of folate, 13.2% of iron, 8.9% of calcium, and 7.5% of magnesium daily. Breads, rolls, and tortillas further contributed 16.6% of thiamin, 10.2% niacin, 7.0% riboflavin, and 6.0% of zinc per day.

### 3.3. Ready-to-Eat Cereals: Sources of Energy and Nutrients by Gender

Ready-to-eat cereals contributed 2.4% of total energy in the U.S. adult female diet (Figure 4). Taking into account dietary guidance to limit sodium, sugar, and fat in the diet, ready-to-eat cereals contributed 1.8% of sodium, 2.6% of total sugar, 0.9% of total fat, and 0.6% of saturated fat daily. Ready-to-eat cereals proved to be an important source for shortfall nutrients in the American diet, with approximately 5.0% of total dietary fiber, 11.3% of folate, 10.6% of iron, and 4.9% of vitamin D contributed daily. Additionally, ready-to-eat cereals provided 9.0% of vitamin B12, 8.8% of vitamin B6, 7.7% of thiamin, 7.2% niacin, 6.9% vitamin A, 6.1% riboflavin, and 6.1% of zinc per day.

For adult males (Figure 5), ready-to-eat cereals contributed 1.7% of sodium, 2.7% of total sugar, minimal amounts of total and saturated fat and less than 3% of total energy—specifically, 0.8% of total fat and 0.5% of saturated fat in the daily diet, in exchange for 2.1% of total energy. For shortfall nutrients identified by the 2015–2020 DGA [1], ready-to-eat cereals contributed approximately 13.8% of total dietary fiber, 16.1% of folate, 13.2% of iron, 8.9% of calcium, and 7.5% of magnesium daily. Ready-to-eat cereals further contributed 16.6% of thiamin, 10.2% niacin, 7.0% riboflavin, and 6.0% of zinc per day.

### 3.4. Cooked Grains: Sources of Energy and Nutrients by Gender

Percentage of total intake of energy and nutrients contributed from cooked grains in adult females and males were minimal relative to other grain products and can be seen in Figure 6 and Figure 7. When considering nutrients to limit in females and males, respectively, cooked grains provided 2.0 and 2.1% of sodium, 0.1 and 0.1% of total sugar, 0.7 and 0.6% of total fat, and 0.4% and 0.4% of saturated fat in the daily diet. Cooked grains provided 1.8 and 1.7% of total energy, 1.3% and 1.4% of dietary fiber, 3.3% and 3.5% of dietary folate, 2.0% and 2.0% of iron, 2.6% and 2.6% of thiamin, 1.7% and 1.6% of niacin, 1.4% and 1.4% of magnesium, 1.4% and 1.3% of zinc and, 1.3% and 1.3% of vitamin B_6_ in females and males, respectively, with negligible contributions of vitamins A, D, B_12_, E, riboflavin, and potassium.

### 3.5. Quick Breads and Bread Products: Sources of Energy and Nutrients by Gender

Percentage of total intake of energy and nutrients contributed from quick breads and bread products for adult females and males can be seen in Figure 8 and Figure 9. When considering nutrients to limit in females and males, the quick breads and bread products category provided less than 5% of all sodium, total sugar, total fat, and saturated fat in the daily diet in both genders. In addition, quick breads and bread products contributed less than 5% of total energy, dietary fiber, folate, iron, thiamin, niacin, magnesium, zinc, vitamin B_6_, vitamin B_12_, vitamin A, riboflavin, vitamin E, and vitamins D.

### 3.6. Sweet Bakery Products: Sources of Energy and Nutrients by Gender

Percentage of total intake of energy and nutrients contributed from sweet bakery products in both genders can be seen in Figure 10 and Figure 11. When considering nutrients to limit in females and males, the sweet bakery grains (i.e., cakes, cookies, pies, etc.) provided meaningful amounts for several nutrients to limit, including total energy, total fat, saturated fat, and total sugar in the daily diet. In addition, sweet bakery grains contributed modestly for nutrients to encourage, and had a smaller contribution to sodium compared with percent of total energy contribution (Females: 4.0% of dietary fiber, 5.2% of folate, 5.7% of iron, 4.2% of thiamin, 2.9% of niacin, 3.3% of riboflavin, and 5.6% of vitamin E, and 3.4% of sodium with lower contributions of magnesium, zinc, potassium and vitamins A, B12, and B6; Males: 4.2% of dietary fiber, 5.2% of folate, 5.7% of iron, 4.2% of thiamin, 2.7% of niacin, 3.2% of riboflavin, and 5.7% of vitamin E, and 3.2% of sodium with lower contributions of magnesium, zinc, potassium, and vitamins A, B_12_, and B_6_).

## 4. Discussion

All grain foods, collectively, in addition to specific grain food groups, are meaningful contributors of nutrient density and provide sources for several shortfall nutrients and nutrients of public health concern (i.e., dietary fiber) as identified by the 2015–2020 DGA. All grain products contributed 14.2% or 285 kcal per day in the total U.S. diet of both genders combined. When considering nutrients to limit as outlined by present and previous dietary guidance [1,10,11,12], grain foods contributed 7.2% total fat, 5.4% saturated fat and 14.6% sodium, and 7.9% total sugar. Similarly, when considering nutrients to encourage (i.e., underconsumed nutrients and nutrients of public health concern), grain foods are meaningful contributors to the daily diet. Additionally, breads, rolls and tortillas, and ready-to-eat cereals are meaningful contributors of dietary fiber, thiamin, folate, iron, zinc, and niacin to the American diet of adults, while certain grain foods alone provide minimal contributions to the diet, including sweet bakery products. Thus, indulgent grains should be consumed in moderation with attention to limiting total caloric intake. Cumulatively, a variety of grain food groups routinely consumed by adults contribute to nutrient density in the total diet and can be encouraged as part of a healthy dietary pattern, while adhering to authoritative recommendations to limit calories, saturated fat, sodium, and added sugar intake.

Our current analyses also assessed the sources of energy and nutrients from specific grain foods, of which included breads, rolls and tortillas, and ready-to-eat cereals. While breads, rolls, and tortillas contributed less than 9% of all sodium, less than 4% of total fat, and less than 3% saturated fat, breads, rolls, and tortillas provided greater than 10% for dietary fiber, folate, and iron in adult men and women. Similarly, ready-to-eat cereals provided minimal daily contributions of energy (<3%), sodium (<2%), total sugar (<3%), total fat (<1%), and saturated fat (<1%), but meaningful contributions of folate, iron, magnesium, thiamin, vitamin B_12_, vitamin A, vitamin D, vitamin E, niacin, and zinc relative to the daily contribution of energy (calories). Since previous data has established that nearly the entire U.S. population consumes a diet with fewer whole grains than recommended [4], the assumption with the current NHANES analysis is that most of the grains consumed are enriched grain food products. Thus, the nutrient contribution of all whole and refined grain food products, including breads, rolls and tortillas and ready-to-eat cereals, can play a key role in helping American adults meet recommendations for underconsumed nutrients and nutrients of public health concern.

The current adult data are aligned with results found from our data previously published in children and adolescents [5]. In particular, for children and adolescents 2–18 years-old, all grain products contributed 14% or 263 kcal per day. In analyses focused on nutrients to encourage (i.e., underconsumed nutrients and nutrients of public health concern), NHANES 2009–2012 data provided evidence that grain foods were meaningful contributors of nutrient density in the American diet of children and adolescents throughout the various age groups examined. The data also showed breads, rolls and tortillas, and ready-to-eat cereals to be meaningful contributors of several nutrients, including dietary fiber, thiamin, folate, iron, zinc, and niacin to children and adolescents. The research concluded that a variety of grain food groups consumed by American children and adolescents contribute to nutrient density in the total diet and should be encouraged as part of a healthy dietary pattern.

It has been recently stated that food processing as an industry may be the stepping stone to urbanization [13]. However, in recent years, a negative perception of processed foods, including many foods within the grains food group, have been misperceived as non-nutrient dense foods that are not compatible within a healthy dietary pattern. Indeed, in recent years processed foods have been reformulated to be lower in total and saturated fat, reduced calories, less sodium, and added sugars [14]. A previous NHANES analysis evaluating the contribution of processed foods to nutrients to encourage and nutrients to limit, as recommended by dietary guidance over a 30-year period in individuals ≥2 years-old, found that energy and sodium intakes significantly increased since the 1970s, but have remained constant since the 1980s and 1990s, while saturated fat, measured as grams per day or as a percentage of calories, significantly declined from the late 1970s to the 2000s [13].

Consumers may also be questioning nutrient bioavailability and the public health value of enrichment and/or fortification practices. The 1998 mandatory folic acid fortification of cereal grain products labeled as enriched in the U.S. contributed to a 36% reduction in neural tube defects from 1996 to 2006 and prevented an estimated 10,000 neural tube defect-affected pregnancies, leading the Centers for Disease Control and Prevention to identify folate fortification as one of the top ten public health achievements in the U.S. [15]. Similarly, an expert working group in the U.S. and Canada found that wheat flour fortification which provided an additional intake of approximately 100–150 µg/day of folic acid significantly lowered prevalence of neural tube defects at birth. The report also found that most adverse effects associated with folic acid overconsumption in adults was related to supplement use and not mandatory food fortification [16]. The International Life Sciences Institute North American committee on Fortification in collaboration with the American Society of Nutrition indicated that future directions in discretionary fortification requires monitoring across all age groups and genders, particularly individuals who are more likely to consume both fortified foods and supplements [17]. Micronutrient malnutrition can have adverse outcomes on health, even at moderate levels of deficiency and in industrialized countries, like the U.S., factors that contribute to reducing the risk and prevalence of micronutrient malnutrition includes greater access to nutrient-rich and fortified foods, high-quality health services and higher income levels [18]. Indeed, food fortification has a long history in the U.S. and other industrialized countries, and provides several advantages when properly administered. Food fortification of commonly-consumed and widely-available foods, such as grain-based foods, have the potential to improve the nutritional status of a substantial portion of the population, independent of income status, with the caveat that fortified foods are included as part of a healthy dietary pattern [15]. This implies that fortified foods play an important role in the American diet, provided that dietary recommendations are met. Importantly, all grain food consumption, whether whole or enriched grains, should be incorporated as part of a recommended dietary pattern. Dietary guidelines promote consuming healthy eating patterns at appropriate calorie levels for age and gender, of which includes choosing a variety of nutrient-dense foods across and within all food groups in recommended amounts. Dietary guidance also promotes increasing whole grains, fruits and vegetables, low-fat dairy foods, and lean protein foods while limiting added sugars, saturated fat, sodium, and total energy [1].

Previous work using NHANES 2003–2006 has examined the nutrient contribution of processed foods, where nutrients were added via enrichment (i.e., replacing nutrient in the food lost during processing) or fortification (i.e., adding nutrients to the food at higher levels than naturally present). Enriched foods included grain products, with particular focus on breads, while fortified foods included ready-to-eat cereals (i.e., fortified with folate, iron, and other nutrients) and milk (i.e., fortified with vitamins A and D). The researchers found that if enrichment and fortification practices were not utilized in the food supply, large percentages of the American population would have inadequate intakes of vitamin A, vitamin C, vitamin D, vitamin E, thiamin, folate, calcium, magnesium, and iron. In contrast, when nutrients from enrichment and fortification were added to the dietary analysis, the percentages of the population with inadequate intakes was meaningfully decreased for vitamin A, vitamin D, folate, and iron [19]. Likewise, an analysis with 2007–2010 NHANES data to model the potential impact of a lack of fortification on overall dietary intake in children and adolescents (2–18 years-old) and adults (19–99 years-old) verified the important role ready-to-eat cereal fortification provides to help achieve nutrient recommendations. Among children and adolescents with intake below the estimated average requirement (EAR), significant nutrient increases resulting from consumption of fortified ready-to-eat cereals ranged from 3.3% for vitamin D (D_2_ + D_3_) to 161.5% for folate. There were also significant increases in iron, thiamin, riboflavin, vitamin A, vitamin B6; vitamin E, niacin, and zinc. Similarly, in adults, significant percentage increases for nutrients below EAR ranged from 8.3% for magnesium to 84.8% for folate. Iron, riboflavin, thiamin, vitamin A, vitamin B_12_, vitamin B_6_, zinc, niacin, and vitamin E also significantly increased as a result of ready-to-eat cereal fortification [16]. Previous work in Americans aged ≥2 years-old, identified significant amounts of vitamins A, B_6_, B_12_, C, and D, as well as thiamin, riboflavin, niacin, folate and iron were from enriched and/or fortified foods, suggesting that without enrichment and/or fortification, nutrient recommendation shortfalls may be further exacerbated [20], further providing evidence to advocate for the beneficial role processed foods can play in promoting public health. Data from the National Health and Nutrition Examination Survey (NHANES) 2003–2006, showed fortification of grain foods provides nutrient adequacy for U.S. children and adolescents, without concern for excessive intakes for most vitamins and minerals [21]. Others have shown that without fortification and enrichment, 20% of the U.S. population would fall below the EAR for iron, with this percentage being lowered to approximately 6% when including enriched and fortified foods [20]. The most robust effects of enrichment and fortification practices have been documented with thiamin and folic acid, nutrients typically added to grain foods through enrichment and fortification practices. Data have shown that without enrichment and fortification, 50% of the U.S. population would have inadequate intakes compared with only 5% when enrichment and fortification are included. Similarly, for folic acid, when not considering mandatory and discretionary enrichment and fortification, nearly 90% of Americans would have inadequate intake versus only 10% with enriched and fortified food consumption [14,21]. Thus, fortified and enriched foods [19,20,21,22], including the grain food category, are a key component of healthy dietary patterns and may provide indispensable benefits to eating patterns by helping individuals attain dietary adequacy. 

The current analyses have limitations inherent within observational research and have previously been documented. The results are dependent on self-reported dietary data for foods, which may involve study participants under- or over-estimating food consumption, leading to inaccuracies in energy and nutrient intakes. Data were also obtained using a 24-h dietary recall, which relies on study participant memory and while validated methods are used to gather the data, recall information is subject to inaccuracies and bias from memory challenges and other potential measurement errors experienced in epidemiological investigations using large datasets [23]. Caution should be administered when comparing the current findings to previous studies as food groupings can impact sources of energy and nutrient outcomes, specifically where there are differences in the level of aggregation (i.e., the number of food groups) or disaggregation methods used by researchers. A significant benefit of using NHANES data for the current analyses includes access to a large and nationally representative dataset of adults in the U.S. and corresponding food, energy and nutrient intake data.

## 5. Conclusions

The present data from a U.S. nationally representative sample of free-living adult females and males demonstrates that cumulatively, a variety of whole and refined/enriched grain food groups consumed, help contribute to nutrient density in the total diet. While all grain foods are contributors of energy, total fat and sodium in the U.S. diet, grain foods are also contributors of folate and iron (2015–2020 DGA underconsumed nutrients) and dietary fiber (2015–2020 DGA nutrient of public health concern) [4]. When considering sub-categories of grain foods, breads, rolls and tortillas, and ready-to-eat cereals are meaningful contributors (i.e., ≥10% in the diet) of dietary fiber, thiamin, folate, iron, zinc and niacin to the American diet of adults. Certain grain foods, as part of healthy dietary patterns that include a selection of enriched and fortified grains, may improve overall nutrient intakes and minimize gaps in shortfall nutrient intakes. In contrast, eliminating grains, including both whole and enriched grains from the diet may lead to unintended nutrient consequences, which may negatively impact public health initiatives and health outcomes, particularly if foods substituted into the diet lack nutrient density and promote excess calories. Therefore, dietary strategies that aim to stay within caloric recommendations while monitoring nutrients to limit (i.e., added sugars, sodium and saturated fat) can benefit from the inclusion of several enriched and whole grain food products in the diet. Choosing both whole and refined/enriched grain foods in nutrient-dense forms [1], can help in meeting recommendations for a healthy eating pattern in adult men and women and is aligned with guidance put forth by the 2015–2020 DGA.

## Figures and Tables

**Table 1:** Grain categories identified from the National Health and Nutrition Examination Survey (NHANES) 2009–2012 in adults (≥19 years-old; *n* = 10,697; gender combined) by mean (SE) gram weight and percent weight (SE) in total diet.

Grain Food Group Category	Mean Weight (g) Consumption (SE)	Percent (%) Weight Consumption in the Total Diet
All Foods	3555 ± 36	100 ± 0
All Grains	126.3 ± 3.0	4.1 ± 0.123
Breads, Rolls & Tortillas	55.7 ± 1.1	1.8 ± 0.03
Ready-to-Eat Cereals	12.2 ± 0.4	0.39 ± 0.01
Cooked Grains	24.6 ± 1.7	0.82 ± 0.06
Quick Breads & Bread Products	12.3 ± 0.7	0.40 ± 0.03
Sweet Bakery Products	36.0 ± 0.84	1.1 ± 0.03
